# Effects of Bisphosphonates Treatments in Osteopenic Older Women: A Systematic Review and Meta-Analysis

**DOI:** 10.3389/fphar.2022.892091

**Published:** 2022-05-19

**Authors:** Jiangbi Li, Yang Sun, Zhuo Chen, Xiaoping Xie, Feng Gu, Songqi Bi, Tiecheng Yu

**Affiliations:** ^1^ Department of Orthopedics, First Hospital of Jilin University, Changchun, China; ^2^ School of Foreign Language, Northeast Normal University, Changchun, China

**Keywords:** osteopenia, bisphosphonates, bone mineral density, fracture, bone markers

## Abstract

**Aims:** To review the effects of bisphosphonates on bone density, fractures, and bone markers in osteopenic older women.

**Methods:** Relevant articles published before February 2022 were searched in PubMed, EMBASE, and the Cochrane Library. All randomized controlled trials that reported incident fractures, bone mineral density (BMD), bone markers, or adverse events with bisphosphonates in osteopenic older women were included. The quality of included studies was assessed using the Cochrane Risk of Bias tool. The risk ratios (RRs) for fractures, net percent change in bone mineral density and differences in bone markers were calculated using a meta-analysis.

**Results:** A total of 11 studies were included in our meta-analysis. Bisphosphonates significantly increased the percent changes in the lumbar spine BMD (WMD, 5.60; 95% CI, 4.16–7.03; *I* ^2^ = 93.6%), hip BMD (WMD, 4.80; 95% CI, 2.93 to 6.66; *I* ^2^ = 97.1%), total body BMD (WMD, 3.24; 95% CI, 2.12–4.35; *I* ^2^ = 90.9%), femoral neck BMD (WMD, 4.02; 95% CI, 1.70–6.35; *I* ^2^ = 91.8%) and trochanter BMD (WMD, 5.22; 95% CI, 3.51–6.93; *I* ^2^ = 83.6%) when compared to placebo. Zoledronate was associated with a great treatment effect on fragility fracture (RR, 0.63; 95% CI, 0.50–0.79), clinical vertebral fracture (RR, 0.41; 95% CI, 0.22–0.76), and radiographic vertebral fracture (RR, 0.60; 95% CI, 0.27–1.35) compared to placebo. Meanwhile, alendronate was also associated with beneficial effects on fragility fracture (RR, 0.40; 95% CI, 0.15–1.07), clinical vertebral fracture (RR, 0.46; 95% CI, 0.17–1.24), and radiographic vertebral fracture (RR, 0.64; 95% CI, 0.38–1.09). In addition, the use of bisphosphonates reduced the concentration of procollagen type I N-terminal propeptide (PINP) and C-terminal telopeptide of type I collagen (CTX) over placebo by 15.79 (95% CI, −18.92 to −12.66; *I* ^2^ = 28.4%), −0.23 (95% CI, −0.35 to −0.10; *I* ^2^ = 91.3%), respectively. Although there was insufficient evidence to determine their safety, these bisphosphonates may have an effect on cancer, cardiac events, and mortality in osteopenic older women.

**Conclusion:** All bisphosphonates examined were associated with beneficial effects on fractures, BMD, and bone markers in women with osteopenia. Further randomized controlled trials are necessary to clarify the safety of bisphosphonates in women with osteopenia.

## 1 Introduction

Osteoporosis is defined as having a bone-density T score of less than 2.5 or having a high rate of vertebral fractures (Kanis et al., 1994). Bisphosphonates, which have been shown to lower fracture risk and enhance bone mineral density, are the most common therapy for osteoporosis (BMD) (Sanderson et al., 2016). However, their efficacy in women with osteopenia, which is defined by a T score of −1.0 to −2.5 (Kanis et al., 1994), has not been shown most clearly. Surprisingly, the vast majority of osteoporotic fractures occur in people with a BMD T score in the osteopenic range (−2.5 <T score < −1). the fact that osteopenia is associated with a lower risk of fracture than osteoporosis, osteopenia affects significantly more people than osteoporosis. (Eriksen, 2012). T scores of greater than 2.5 were seen in about 82% of postmenopausal women with fractures (Siris et al., 2004). According to the findings of Pasco et al. (2016), 37.6% of the women had normal total hip BMD, 48.0% had osteopenia, and 14.5% had osteoporosis. Women with osteoporosis had the highest rate of fracture throughout follow-up, although only 26.9% of total fractures occurred in this group, whereas 56.5% occurred in women without osteopenia (Pasco et al., 2006). Based on the overall 43.9% prevalence of osteopenia, 43.4 million older adults were estimated to have osteopenia in 2010 (Wright et al., 2014). The majority of fractures in the population are caused by osteopenia, not osteoporosis. Fractures caused by osteoporosis, such as vertebral and hip fractures, can increase morbidity and mortality, as well as treatment costs (Cummings and Melton, 2002). As a result, effective treatments for women with osteopenia are needed to keep the low bone mass from progressing to osteoporosis.

Recently, several clinical trials evaluating bisphosphonate treatments in women with osteopenia have been reported. To the best of our knowledge, no meta-analysis of such studies has been carried out. We conducted a comprehensive review and meta-analysis of the bisphosphonates’ efficacy in women with osteopenia. We attempted to include all published randomized control studies that assessed the effects of bisphosphonates on bone mineral density (BMD), incident fractures, bone markers, or adverse events in women with osteopenia.

## 2 Methods

The Preferred Reporting Items for Systematic Reviews and Meta-analyses guidelines (Liberati et al., 2009) were used to present this meta-analysis.

### 2.1 Search Strategy

Li and Sun, two independent reviewers, conducted a systematic search of PubMed, EMBASE, and the Cochrane Library for relevant papers published before February 2022. The search terms included “alendronate,” “risedronic acid,” “ibandronic acid,” “zoledronic acid,” “etidronic acid,” “clodronic acid,” “pamidronate,” “tiludronic acid,” “6-amino-1-hydroxyhexane-1,1-diphosphonate” and “osteopenia,” “osteopenias,” “low bone density,” “bone density, low,” “low bone densities” and “bone density,” “fractures, bone,” “bone markers,” “adverse effects.” Supplementary Table S1 summarizes the search techniques in detail. By checking through the references of relevant research and review publications, additional studies were discovered.

### 2.2 Selection Criteria

Studies were considered eligible if they met the following criteria: 1) it was a randomized controlled trial; 2) it included patients with osteopenia (defined by a T score of −1.0 to −2.5 at the lumbar spine, hip, or femoral neck); 3) had compared alendronate, risedronate, ibandronate, zoledronate, etidronate, clodronate, pamidronate, tiludronate, or neridronate with placebo; 4) had evaluated bone mineral density (BMD), fractures, bone markers, or adverse events; 5) all studies had to have followed at least 20 patients for at least 12 months.

The following were exclusion criteria: 1) duplicate articles; 2) reviews, case reports, letters, editorials, and meta-analyses; and 3) molecular biology or animal studies. After deleting duplicate articles, two investigators (Li and Sun) independently reviewed the articles by title and abstract. The full texts were then retrieved to identify the appropriate papers. Disagreements in study selection were resolved through detailed discussion or consultation when necessary. When duplicate studies were identified, only the most complete and recent study data were considered.

### 2.3 Data Extraction and Quality Assessment

For each study, the first author’s name, publication year, study design, country, treatments and co-interventions, sample size, age, BMD T-score, follow-up period, and reported outcomes, including measures of variability, were retrieved. Reported outcomes from the last time point of the study were extracted. If standard deviations were not reported, we calculated the standard deviation using confidence intervals. To extract data simply displayed in figures that did not match numeric data, we used image extraction software (Engauge Digitizer). We assessed the quality of included studies using the Cochrane Risk of Bias Tool (Higgins and Thomas, 2021). Data extraction and quality assessment were performed independently by two authors (Li and Sun).

### 2.4 Statistical Analysis

Analyses were performed using Stata 12.0 software. In the meta-analysis for BMD outcomes, we used the reported or calculated net percent difference between the diphosphonate and placebo groups as a measure of effect size because most RCTs provided within-group percent changes in BMD outcomes. The fracture with bisphosphonate use was measured by a summary risk ratio (RR) with a 95% confidence interval (CI) derived from HRs and ORs. Because fractures are rare, the OR is an approximation of the relative risk of fracture. The reported or calculated change (difference in the two within-group changes from baseline) between the diphosphonate and placebo groups was used as a measure of effect size in the meta-analysis for the PINP and CTX outcomes. When we concluded that the data from at least two studies were sufficiently homogeneous, we performed meta-analyses. The statistics *I* ^2^ and *Q* were used to assess the heterogeneity of the studies. Because *I* ^2^ > 50% and *p* < 0.05 indicated substantial heterogeneity between the studies examined, a random-effect model was used to pool the data; otherwise, a solid effect model was used. To examine the robustness of the results, sensitivity analyses were performed by eliminating each included paper, and publication bias was assessed using the Begg and Egger test.

## 3 Results

### 3.1 Search Results

**FIGURE 1:**
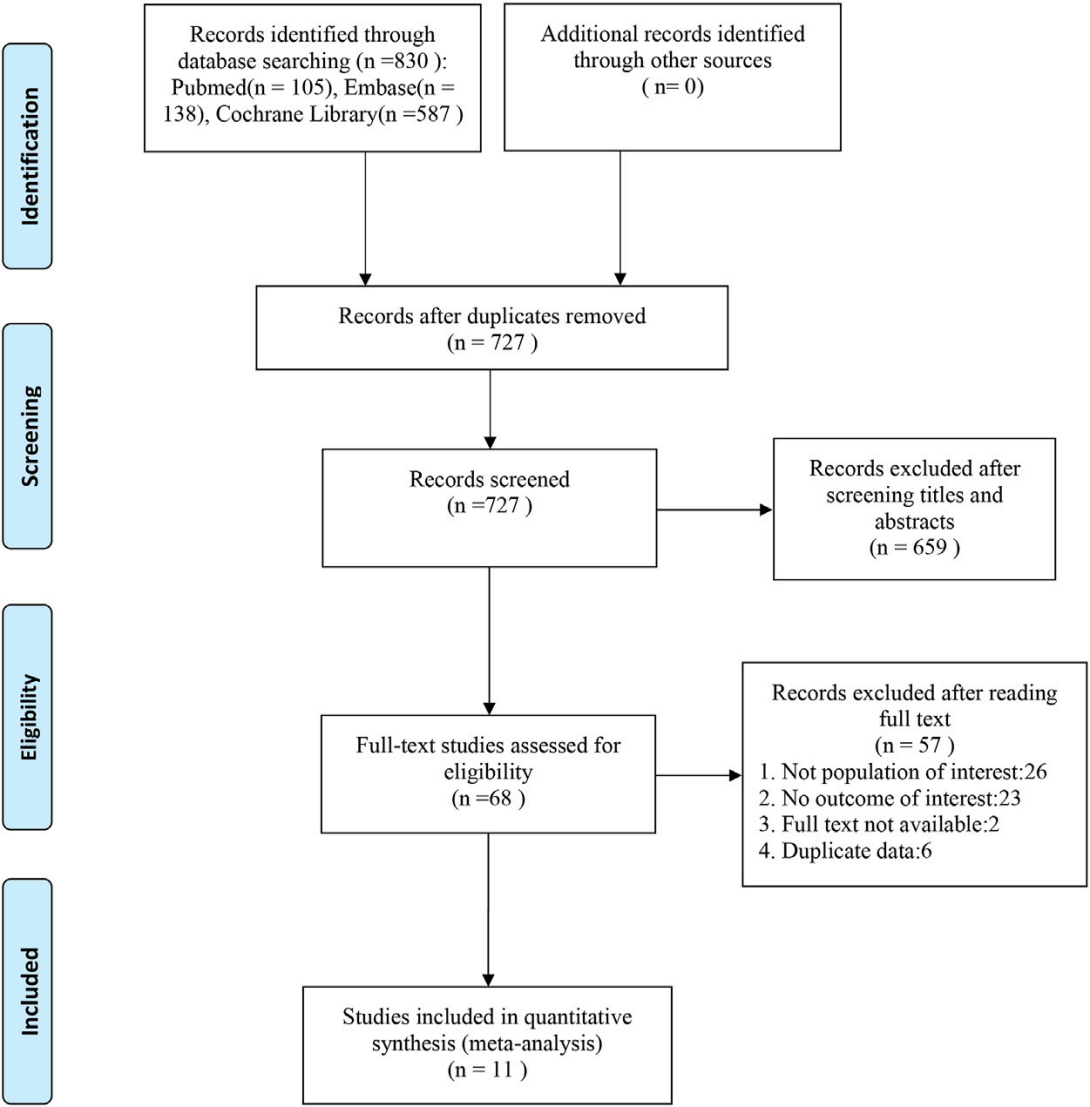
Selection of studies for inclusion.

We initially identified 830 potentially eligible studies after the literature search process. After removing duplicates from the 830 papers found, 727 remained, of which 68 were selected as possibly suitable after examining the titles and abstracts. After reviewing the abstracts and full texts, we included 11 studies that evaluated a bisphosphonate in terms of BMD, fractures, bone markers, or adverse events among a total of 7,114 patients with osteopenia. Finally, 11 studies were found to be eligible for inclusion in our meta-analysis. The literature search process is illustrated in Figure 1.

### 3.2 Characteristics of Included Studies

In our meta-analysis, 11 (Yen et al., 2000; McClung et al., 2004; Quandt et al., 2005; Välimäki et al., 2007; McClung et al., 2009a; McClung et al., 2009b; Grey et al., 2012; Grey et al., 2017; Reid et al., 2018; Sestak et al., 2019; Zhou et al., 2020) studies were considered. Table 1 shows the detailed features of the included studies. They were all randomized controlled trials published between 2000 and 2020. Four studies were conducted in the USA, three in New Zealand, two in Europe, one in China. Ten studies reported BMD, four studies reported PINP and CTX, and three studies reported fractures. Besides, four studies evaluated zoledronate, three studies evaluated alendronate, two studies evaluated ibandronate, and two studies evaluated risedronate. In addition, eight studies reported adverse events. In most trials, patients were given calcium or vitamin D supplements, or both, at the same time. The participants in all of the trials were adult women ranging in age from 53–84 years old. The study duration ranged from 1–6 years.

**TABLE 1:** Characteristics of 11 included studies.

Author (Year)	Study Design	Site	Intervention	Co-interventions	No. of Participants(Treatment/Control)	Mean Age (Year) (Treatment/Control)		BMD T- score (Treatment/Control)		Duration	Reported Outcomes	Risk of Bias
McClung et al. (2009a)	RCT	United States	zoledronic acid 2x5 mg vs. placebo	500-mg to 1,200-mg elemental calcium and vitamin D 400–800 international units daily	198/202	59.9 ± 8.0	60.5 ± 8.0	1.67 ± 0.42	1.71 ± 0.46	2 year	BMD, bone markers,adverse event	Low risk
Grey et al. (2012)	RCT	New Zealand	iv. zoledronate 5 mg vs placebo	—	20/21	62 (8)	67 (8)	−1.0 (0.8)	−1.2 (0.7)	5 year	BMD, bone markers, adverse event	Low risk
Grey et al. (2017)	RCT	New Zealand	iv.zoledronate 5 mg vs. placebo	—	41/34	66 ± 8	63 ± 8	–1.1 ± 1.0	–1.4 ± 0.8	5 year	BMD, bone markers	Low risk
Reid et al. (2018)	RCT	New Zealand	iv.zoledronate 5 mg/18 months vs. control	1.25 mg cholecalciferol/month required, 1g calcium advised	1000/1000	71±5.0	71±5.1	−1.27±0.59	−1.24±0.60	6 year	BMD, fracture, bone markers, adverse event	Low risk
Yen et al. (2000)	RCT	China	oral alendronate 10 mg/d vs placebo	500 mg calcium daily	24/22	59 ± 4.7	60.3 ± 6.5	0.72 ± 0.08	0.721 ± 0.08	1 year	BMD, adverse event	Unclear risk
Quandt et al. (2005)	RCT	United States	alendronate 5 mg/d for 2 years and 10mg/d for another 2.5 years vs placebo	500 mg elemental calcium and 250IU cholecalciferol daily	1878/1859	67.6	67.8	-2.5<T<−1.6	-2.5<T<−1.6	4.5 year	fracture	Unclear risk
Zhou et al. (2020)	RCT	China	oral alendronate 70 mg/week vs. control	600 mg/d of calcium carbonate and 0.5 μg/d of alfacalcidol	62/61	83.16 ± 3.09	83.92 ± 2.85	−2.5<T<−1	-2.5<T<−1	18 months	BMD, fracture, bone markers, adverse event	High risk
Valimaki et al. (2007)	RCT	Finland	risedronate 5 mg/d vs placebo	1000 mg of elemental calcium and 400 IU of vitamin D daily	114/56	66.1 (6.8)	65.4 (6.8)	−1.81 (0.41)	-1.84 (0.44)	2 year	BMD, adverse event	Unclear risk
Sestak et al. (2019)	RCT	United Kingdom	oral risedronate 35 mg/week vs. placebo	vitamin D and calcium (advised, but not required)	59/74	60.8 (7.67)	59.7 (12.5)	−2.5<T<−1	-2.5<T<−1	5 year	BMD, adverse event	Lo w risk
McClung et al. (2004)	RCT	United States	oral ibandronate 2.5 mg/d vs placebo	calcium (500 mg daily)	106/102	58.2 ± 8.6	57.9 ± 8.6	0.93 ± 0.05	0.92 ± 0.05	2 year	BMD	Unclear risk
McClung et al. (2009b)	RCT	United States	oral ibandronate 150 mg/month vs placebo	calcium (500 mg/day) and vitamin D (400 IU/day)	77/83	53.7 ± 3.6	53.4 ± 3.8	−1.6 ± 0.4	−1.6 ± 0.4	1 year	BMD, adverse event	Unclear risk

### 3.3 Risk of Bias

We assigned studies a low, uncertain, or high risk of bias (Supplementary Table S2). Five studies have a low risk of bias, five have an unknown risk of bias, and just one has a high risk of bias. Only one of the 11 studies stated that participants and study workers were not blinded (Zhou et al., 2020). The total studies that compared zoledronate with placebo have a low overall risk of bias, and the effects of the zoledronate on BMD, fractures, and bone markers in women with osteopenia were consistent (Supplementary Table S3). Some studies did not provide enough information on sequence generation or allocation concealment, or they reported insufficient results.

### 3.4 Random Effects Meta-Analysis: Bone Mineral Density

#### 3.4.1 Percent Change of Lumbar Spine BMD

**FIGURE 2:**
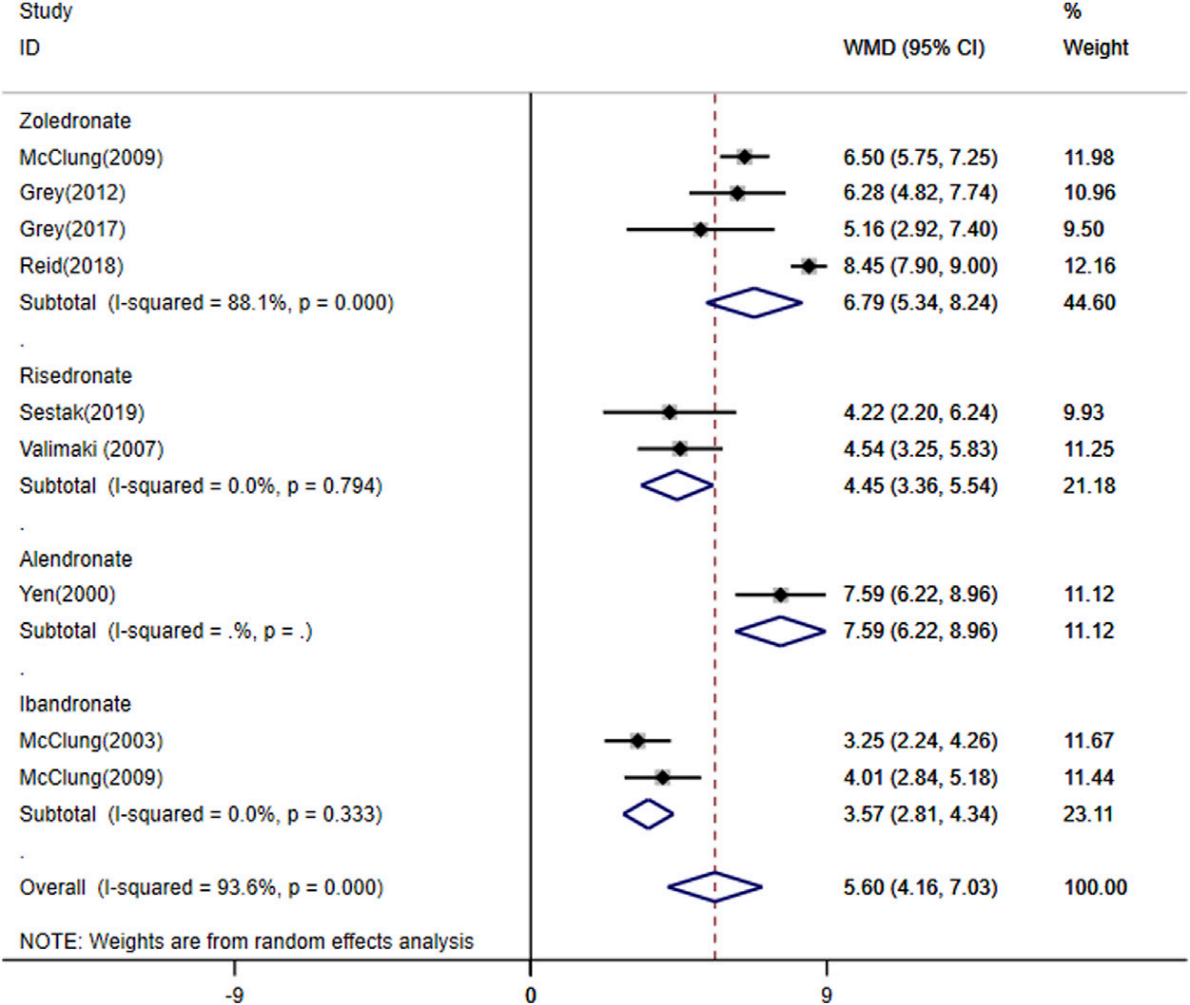
Meta-analysis of the effects of bisphosphonates on lumbar spine BMD.

Bisphosphonate was compared to a placebo in ten randomized controlled trials (RCTs). As shown in Figure 2, a total of four bisphosphonates increased the percent change of spine BMD over placebo by 5.60 (95% CI, 4.16–7.03; *I* ^2^ = 93.6%). Besides, zoledronate, ibandronate, risedronate and alendronate increased the percent change of spine BMD over placebo by 6.79 (95% CI, 5.34 to 8.24; *I* ^2^ = 88.1%), 3.57 (95% CI, 2.81 to 4.34; *I* ^2^ = 0.0%), 4.45 (95% CI, 3.36–5.54; *I* ^2^ = 0.0%), 7.59 (95% CI, 6.22–8.96), respectively. Bisphosphonates are the source of heterogeneity. The bisphosphonate types, which decreased by 93.6% in the group with ibandronate and risedronate, contributed to the heterogeneity. The *p*-value for the publication bias evaluated by the Begg’s test and Egger’s test was 0.917 and 0.076, respectively.

#### 3.4.2 Percent Change of Hip BMD

**FIGURE 3:**
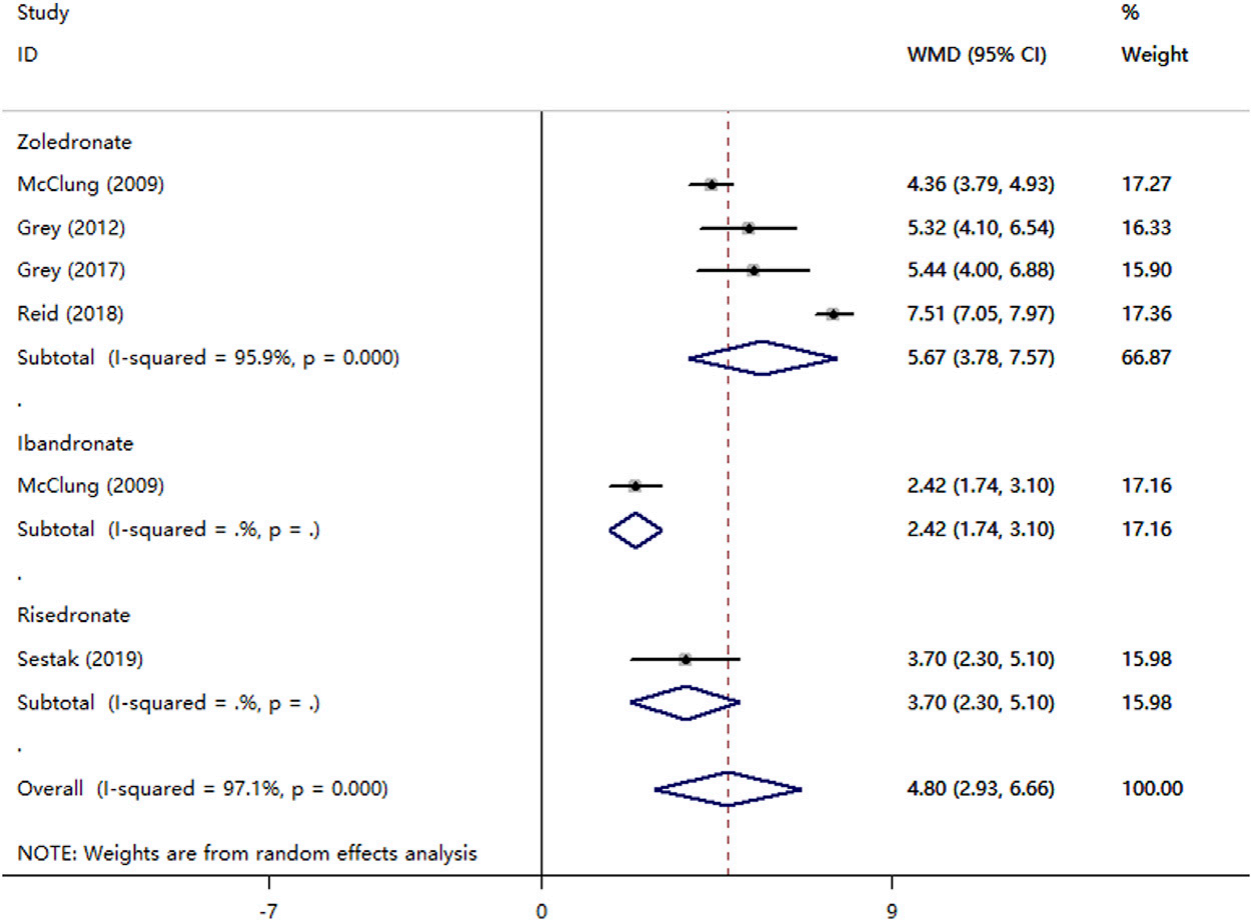
Meta-analysis of the effects of bisphosphonates on hip BMD.

Six RCTs compared bisphosphonate with a placebo. Figure 3 shows that a total of six bisphosphonates increased the percent change of hip BMD over placebo by 4.80 (95% CI, 2.93–6.66; *I* ^*2*^ = 97.1%). Besides, zoledronate, ibandronate, and risedronate increased the percent change of hip BMD over placebo 5.67 (95% CI, 3.78–7.57; *I* ^*2*^ = 95.9%), 2.42 (95% CI, 1.74–3.10), 3.7 (95% CI, 2.30–5.10; *I* ^*2*^ = 0.0%), respectively. The *p*-value for the publication bias evaluated by the Begg’s test and Egger’s test was 1.0 and 0.486, respectively.

#### 3.4.3 Percent Change of Trochanter BMD

Three RCTs compared bisphosphonate with placebo in the meta-analysis (Supplementary Figure S1A). A total of three bisphosphonates increased the percent change of trochanter BMD by 5.22 (95% CI, 3.51–6.93; *I* ^*2*^ = 83.6%) as compared to a placebo. Additionally, zoledronate, ibandronate, and alendronate increased the percent change of trochanter BMD over placebo by 5.98 (95% CI, 5.22–6.74), 3.78 (95% CI, 2.76–4.80), 6.23 (95% CI, 3.86–8.60), respectively. The *p*-value for the publication bias evaluated by the Begg’s test and Egger’s test was 1.0 and 0.955, respectively.

#### 3.4.4 Percent Change of Femoral Neck BMD

Three RCTs compared bisphosphonate with placebo in the meta-analysis. (Supplementary Figure S1B). Three bisphosphonates increased the percent change of femoral neck BMD by 4.02 (95% CI, 1.70–6.35; *I* ^2^ = 91.8%) as compared to a placebo. In addition, zoledronate, ibandronate, and alendronate increased the percent change of femoral neck BMD over placebo by 3.55 (95% CI, 2.74–4.36), 1.84 (95% CI, 0.76–2.92), 7.02 (95% CI, 5.27–8.77), respectively. The *p*-value for the publication bias evaluated by the Begg’s test and Egger’s test was 1.0 and 0.548, respectively.

#### 3.4 5 Percent Change of Total Body BMD

Four RCTs compared results for participants receiving zoledronate vs placebo (Supplementary Figure S1C). Our meta-analysis of four RCTs showed that zoledronate increased the percent change of total body BMD over placebo by 3.24 (95% CI, 2.12–4.35; *I* ^2^ = 90.9%). The *p*-value for the publication bias evaluated by Begg’s test and Egger’s test was 1.0 and 0.607, respectively.

### 3.5 Random Effects Meta-analysis: Fracture

**FIGURE 4:**
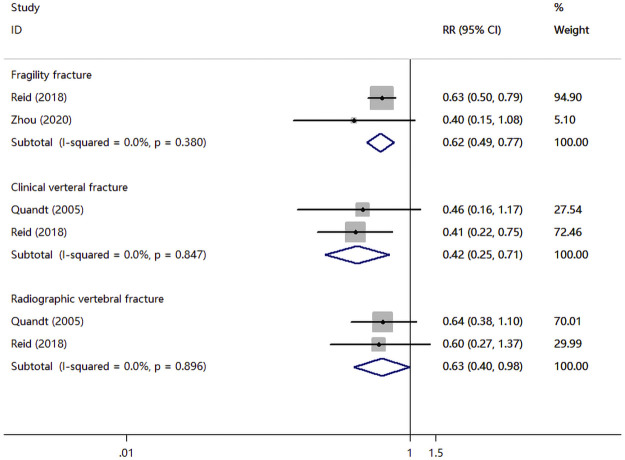
Meta-analysis results of bisphosphonates for the incidence of fragility fracture, clinical verteral fracture, and radiographic vertebral fracture.

Three RCTs compared zoledronate or alendronate with a placebo. As shown in Figure 4, There was significant association of zoledronate with fragility fracture (RR, 0.62; 95% CI, 0.49 to 0.77; *I* ^2^ = 0.0%), clinical verteral fracture (RR, 0.42; 95% CI, 0.25 to 0.71; *I* ^2^ = 0.0%), radiographic vertebral fracture (RR, 0.63; 95% CI, 0.40–0.98; *I* ^2^ = 0.0%).

### 3.6 Random Effects Meta-Analysis: Bone Markers

**FIGURE 5:**
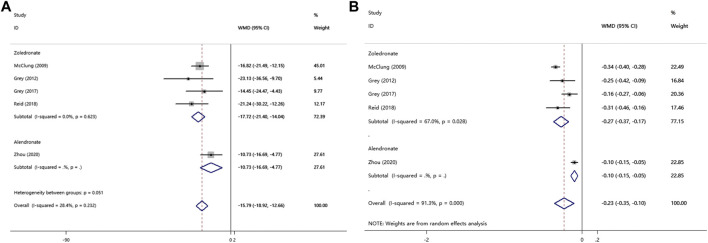
Meta-analysis of the effects of bisphosphonates on **(A)** PINP and **(B)** CTX.

Five RCTs examined PINP and CTX levels of patients. As shown in Figure 5A, a total of four bisphosphonates reduced the levels of PINP over placebo by −15.79 (95% CI, −18.92 to −12.66; *I* ^2^ = 28.4%). Besides, zoledronate and alendronate reduced PINP over placebo by −17.72 (95% CI, −21.40 to −14.04; *I* ^2^ = 0.0%), −10.73 (95% CI, −16.69 to −4.77), respectively. The *p*-value for the publication bias evaluated by the Egger test was 0.492. Meanwhile, five bisphosphonates reduced the levels of CTX over placebo by −0.23 (95% CI, −0.35 to −0.10; *I* ^2^ = 91.3%) (Figure 5B). In the group with zoledronate, CTX was remarkably reduced when compared to controls (WMD, −0.27; 95% CI, −0.37 to −0.17). Besides, a significant difference for CTX was observed when comparing women with alendronate to women with control in those with osteopenia (WMD, −0.10; 95% CI, −0.15 to −0.05). The *p*-value for the publication bias evaluated by Begg’s test and Egger’s test was 0.806 and 0.629, respectively.

### 3.7 Risk Differences in Adverse Events

Table 2 summarized the risk differences (RDs) in adverse events. Eight trials reported adverse events. Six studies reported gastrointestinal adverse events, with rates ranging from −7.2% to 8.1%. However, none of these trials was powered to detect a difference in gastrointestinal adverse events. One trial (Reid et al., 2018) found that there was a difference between zoledronate with control in the rate of death events (2.7% vs. 4.1%), cancer events (8.4% vs. 12.1%), composite of vascular events (5.3% vs. 6.9%) and myocardial infarction (2.4% vs. 3.9%). respectively. Three trials (Yen et al., 2000; Grey et al., 2012; Zhou et al., 2020) reported hypercalcemia, with rates ranging from 0 to 1.6%. Two trials (McClung et al., 2009a; McClung et al., 2009b) reported no statistically significant differences in musculoskeletal pain, nausea, and arthralgia between the treatment and control groups (RD range, −0.5%–15.7%). Besides, the other two trials (Grey et al., 2012; Sestak et al., 2019) reported no osteonecrosis of the jaw events in the bisphosphonates and placebo group.

**TABLE 2:** Summary of the number of studies (number of participants, risk difference) and the range in risk difference in adverse events in randomized, controlled trials.^a^

Adverse events	Zoledronate vs. placebo	Risedronate vs. placebo	Alendronate vs. placebo	Ibandronate vs. placebo
Gastrointestinal adverse events	0 studies	1 (170, −7.2%)	1 (123, 8.1%)	1 (160, 6.2%)
Death	1 (2000, −1.4%)	0 studies	0 studies	0 studies
Hypercalcemia	1 (50, 0%)	0 studies	2 (169.0% to 1.6%)	0 studies
Hypercalciuria	0 studies	0 studies	1 (123, 4.0%)	0 studies
Cancer	1 (2000, −3.7%)	0 studies	1 (123, 0.8%)	0 studies
Infection	1 (383, −4.4%)	0 studies	1 (123,−0.8%)	0 studies
Composite of vascular events	1 (2000, −1.6%)	0 studies	0 studies	0 studies
Atrial fibrillation	1 (2000, −0.1%)	0 studies	0 studies	0 studies
Musculoskeletal pain	1 (383, 15.7%)	0 studies	0 studies	1 (160, 4.1%)
Nausea	1 (383, 3.6%)	0 studies	0 studies	1 (160, 2.8%)
Arthralgia	1 (383, −0.5%)	0 studies	0 studies	1 (160, 5.9%)
Myocardial infarction	1 (2000, −1.5%)	0 studies	0 studies	0 studies
Osteonecrosis of the jaw	1 (41, 0%)	1 (133, 0%)	0 studies	0 studies

a The risk difference was calculated by subtracting the percentage of participants experiencing an adverse event in the placebo or control group from the percentage experiencing an adverse event in the treatment group.

### 3.8 Sensitivity Analyses

To test the results’ robustness, sensitivity analyses were performed. The random effects meta-analysis for bone density, fracture, and bone markers in this meta-analysis often remain stable after eliminating each research at a time (Supplementary Figures S2–S7).

## 4 Discussion

We conducted a systematic review and meta-analysis to synthesize the comparative effectiveness of bisphosphonates in women with osteopenia. These studies show good effects of these medicines in the treatment of osteopenia in women when compared to placebo. The presented meta-analysis provides a richer evidence base for assessing potential treatment effects. Our analysis involves measures in terms of fracture outcomes, BMD, select bone turnover markers, and adverse events. We found that administration of bisphosphonates significantly increased bone BMD over placebo and reduced the risk of fragility fractures and clinical vertebral fractures in women with osteopenia. The concentrations of PINP and CTX were significantly lower in women with osteopenia who received zoledronate or alendronate than in women who received a placebo.

Bisphosphonates are the most commonly prescribed osteoporosis drugs, as they reduce the rate of bone remodeling by suppressing osteoclast activity. The antiresorptive effect of bisphosphonates is determined by their affinity for hydroxyapatite, distribution and duration in bone, and ability to inhibit the enzyme farnesyl pyrophosphate synthase (FPPS) in osteoclasts (Russell et al., 2008). The most commonly used bisphosphonates include orally administered alendronate, risedronate and ibandronate, and intravenously administered zoledronic acid. As shown in a previous meta analysis in women with postmenopausal osteoporosis, zoledronate, ibandronate, risedronate, and alendronate increased spine BMD over placebo by 3.76% (Wang, 2017), 4.80% (Hou et al., 2015), 2.85% (Yang et al., 2019), 7.48% (Cranney et al., 2002a), respectively. Our findings were also consistent with those results and showed that bisphosphonates increased spine BMD in women with osteopenia over placebo from 3.57%–6.20%. For percentage change in the hip, total body, femoral neck, and trochanter BMD, the treatment effects were also statistically significant for all treatments. Osteoporosis treatments-related BMD improvements were significantly linked to fracture reductions (Black et al., 2020). Bisphosphonates substantially reduce the risk of both vertebral and nonvertebral fractures for postmenopausal osteoporosis (Cranney et al., 2002b). At the same time, four RCTs provided fracture data for our analysis, zoledronic acid and alendronate were associated with a great treatment effect on fragility fracture, clinical vertebral fracture and radiographic vertebral fracture for women with osteopenia. Oral bisphosphonates have been shown to result in a magnitude decrease in serum CTX and PINP markers (Black et al., 2007; Bell et al., 2016; Naylor et al., 2016), which are markers of bone formation and resorption, respectively, and they are recommended for monitoring response to bisphosphonate therapy. Bone turnover markers can be used to monitor the individual response of postmenopausal women taking antiresorptive medication (Diez-Perez et al., 2017). Serum PINP, which is primarily derived from bone, rises following bone formation-stimulating treatment. CTX-I is a byproduct of the degradation of type I collagen. They are bone-specific markers that are reduced by antiresorptive medicine (Eastell and Szulc, 2017). Alendronate and zoledronate are oral bisphosphonates that are frequently used to treat osteoporosis. In our meta-analysis, the bone turnover marker (CTX and PINP) was remarkably reduced for alendronate and zoledronate in women with osteopenia. Therefore, CTX and PINP are beneficial in identifying responses to bisphosphonate treatment in postmenopausal osteopenia.

We could not perform a meta-analysis of adverse events in bisphosphonates, because the included studies did not have sufficient data about adverse events. The types of adverse events of bisphosphonates reported vary widely in these articles, which primarily reported on the effectiveness of bisphosphonates, with adverse events as a secondary report. In our included articles, no more than 2 studies reported each adverse event. However, data from at least 3 available studies were required for in our meta-analysis. We did not perform a meta-analysis of adverse events, which was a limitation of our study, and more reports on bisphosphonate adverse events in osteopenic older women are needed in the future. Although we did not perform a meta-analysis of adverse events, we also presented some evidence for adverse effects of the bisphosphonates. Eight trials reported on adverse events found no statistically significant differences in gastrointestinal adverse events, musculoskeletal pain, nausea, and arthralgia between the treatment and control groups. However, One trial (Reid et al., 2018) found that there was a difference between zoledronate with control in the rate of death events, cancer events, composite of vascular events, and myocardial infarction. Another study found that osteopenic older women who were randomly assigned to zoledronate had lower mortality, fewer vascular events, and a lower incidence of cancer (Reid et al., 2020). The phase 3 study of zoledronate for osteoporosis showed a reduction in mortality, which might be attributed to fewer cardiac, respiratory, and neoplastic deaths (Lyles et al., 2007; Colón-Emeric et al., 2010). According to one meta-analysis, effective osteoporosis therapies had lower death rates (Bolland et al., 2010). Several studies have found that bisphosphonates may have anti-cancer effects. Bisphosphonates inhibit the growth of neoplastic cells *in vitro* (Cornish et al., 2011). Bisphosphonates have anticancer effects in animals, lowering tumor burden in bone and non-osseous tissues (Holen and Coleman, 2010). There is clinical trial evidence that bisphosphonates lower the incidence, progression, and death of breast cancer (Early Breast Cancer Trialists' Collaborative Group, 2015). A considerable amount of preclinical and observational research suggests that bisphosphonates lower the risk of vascular disease. A recent meta-analysis of 61 studies in diverse patient groups, including those with osteoporosis and cancer, found that bisphosphonates decreased arterial wall calcification, cardiovascular mortality, and all-cause mortality (Kranenburg et al., 2016). According to one meta-analysis, the usage of bisphosphonates in adult non-cancer patients was associated with an increased incidence of jaw osteonecrosis (OR 2.57; 95% CI 1.37−4.84) (Lee et al., 2014). However, two trials (Grey et al., 2012; Sestak et al., 2019) reported no osteonecrosis of the jaw events in osteopenic older women for bisphosphonates and placebo groups. More studies are needed to confirm bisphosphonates’ effects on cancer, cardiac events, mortality, and osteonecrosis of the jaw in osteopenic older women.

Our meta-analysis has several strengths. Previously, there were many meta-reviews on the efficacy of bisphosphonates for postmenopausal osteoporosis. However, this meta-review was the first to review the efficacy of bisphosphonates in osteopenic older women. In addition, the presented meta-analysis provides richer evidence for assessing the treatment effect of bisphosphonates. Our analysis involves measures in terms of fracture outcomes, BMD, select bone turnover markers, and adverse events. However, there are certain limitations in our meta-analysis. First, the results of our meta-analysis are highly heterogeneous due to differences in research design (criteria for participation, dosing, duration of administration, length of follow-up) and a small number of studies, especially for the primary endpoint, bone fractures. Second, we may have overlooked unpublished trials and those that were not written in English, leading to an overestimation of treatment efficacy. Third, we were unable to do an adverse event meta-analysis since many studies failed to disclose a variety of adverse events.

## 5 Conclusion

In summary, this updated meta-analysis of the randomized controlled trial showed that alendronate, risedronate, ibandronate, zoledronate were all have good therapeutic effects in women with osteopenia. Due to the inherent limitations of this meta-analysis, further large-scale investigations are necessary to corroborate our findings.

## Data Availability

The original contributions presented in the study are included in the article/Supplementary Material, further inquiries can be directed to the corresponding author.
