# Supplementary material for: Effects of Bisphosphonates Treatments in Osteopenic Older Women: A Systematic Review and Meta-Analysis
Source: Front Pharmacol. 2022 May 19;13:892091. doi: 10.3389/fphar.2022.892091 (PMC9160388; doi:10.3389/fphar.2022.892091)

**Supplementary**

**Table S1. Summary of the search strategies.**

**Table S2. Risk of bias of randomized controlled trials evaluating bisphosphonates in women with osteopenia**

**Table S3. Summary of effects of the zoledronate, risedronate, alendronate and ibandronate on BMD, fractures and bone markers in women with osteopenia.**

**Figure S1. Meta-analysis of the effects of bisphosphonates on BMD at three skeletal sites weighted mean difference in (A) trochanter BMD, (B) femoral neck BMD, and (C) total body BMD. BMD: bone mineral density.**

**Figure S2. Sensitivity analysis for the lumbar spine BMD meta-analysis.**

**Figure S3. Sensitivity analysis for the hip BMD meta-analysis.**

**Figure S4. Sensitivity analysis for the trochanter BMD meta-analysis.**

**Figure S5. Sensitivity analysis for the femoral neck BMD meta-analysis.**

**Figure S6. Sensitivity analysis for the total body BMD meta-analysis.**

**Figure S7. Sensitivity analysis for the PINP meta-analysis**

**Figure S8. Sensitivity analysis for the CTX meta-analysis.**

| **Table S1. Summary of the search strategies** | | |
| --- | --- | --- |
| **Item** | **Search terms** | **Results** |
| Pubmed |  |  |
| #1 | ((((((((((((((((((((((((((((((((((((((((((((((((((((((((("**Alendronate**"[Mesh]) OR (4-Amino-1-Hydroxybutylidene 1,1-Biphosphonate[Title/Abstract])) OR (Aminohydroxybutane Bisphosphonate[Title/Abstract])) OR (MK-217[Title/Abstract])) OR (Alendronate Monosodium Salt, Trihydrate[Title/Abstract])) OR (Alendronate Sodium[Title/Abstract])) OR (Fosamax[Title/Abstract])) OR (**Risedronic Acid**[MeSH Terms])) OR (1-Hydroxy-2-(3-pyridyl)ethylidene diphosphonate[Title/Abstract])) OR (Risedronate Sodium[Title/Abstract])) OR (2-(3-pyridinyl)-1-hydroxyethylidenebisphosphonate[Title/Abstract])) OR (Risedronate[Title/Abstract])) OR (Bisphosphonate Risedronate Sodium[Title/Abstract])) OR (Risedronic Acid, Monosodium Salt[Title/Abstract])) OR (**Ibandronic Acid**[MeSH Terms])) ) OR (Ibandronate[Title/Abstract])) OR (1-Hydroxy-3-(methylpentylamino)propylidenebisphosphonate[Title/Abstract])) OR (Ibandronate Sodium Anhydrous[Title/Abstract])) OR (Boniva[Title/Abstract])) OR (RPR 102289A[Title/Abstract])) OR (BM 21.0955[Title/Abstract])) OR (**Zoledronic Acid**[MeSH Terms])) OR (2-(Imidazol-1-yl)-1-hydroxyethylidene-1,1-bisphosphonic acid[Title/Abstract])) OR (CGP 42446A[Title/Abstract])) OR (Zometa[Title/Abstract])) OR (Zoledronic Acid Anhydrous[Title/Abstract])) OR (Zoledronate[Title/Abstract])) OR (**Etidronic Acid**[MeSH Terms])) OR (Hydroxyethylidene Diphosphonic Acid[Title/Abstract])) OR (Etidronate[Title/Abstract])) OR (Hydroxyethanediphosphonate[Title/Abstract])) OR (Etidronate Disodium[Title/Abstract])) OR (Disodium 1-Hydroxyethylene Diphosphonate[Title/Abstract])) OR (HEDSPA[Title/Abstract])) OR (Etidronate, Tetrapotassium Salt[Title/Abstract])) OR (**Clodronic Acid**[MeSH Terms])) OR (Dichloromethane Diphosphonate[Title/Abstract])) OR (Clodronate[Title/Abstract])) OR (Clodronate Disodium[Title/Abstract])) OR (Bonefos[Title/Abstract])) OR (**pamidronate**[MeSH Terms])) OR (Amino-1-hydroxypropane-1,1-diphosphonate[Title/Abstract])) OR (Amidronate[Title/Abstract])) OR (1-Hydroxy-3-aminopropane-1,1-diphosphonic acid[Title/Abstract])) OR (Pamidronate Disodium[Title/Abstract])) OR (**tiludronic acid**[MeSH Terms])) OR ((4-chlorophenyl)thiomethylene bisphosphonic acid[Title/Abstract])) OR (tiludronate disodium[Title/Abstract])) OR (Cl2SMBP[Title/Abstract])) OR (**6-amino-1-hydroxyhexane-1,1-diphosphonate**[MeSH Terms])) OR (neridronate[Title/Abstract])) OR (aminohexane bisphosphonate[Title/Abstract])) OR (6-AHHDP[Title/Abstract])) OR (neridronic acid[Title/Abstract]) | 16284 |
| #2 | (((Osteopenia[Title/Abstract])) OR (Osteopenias[Title/Abstract])) OR (Low Bone Density[Title/Abstract])) OR (Bone Density, Low[Title/Abstract])) OR (Low Bone Densities[Title/Abstract]) | 11253 |
| #3 | (((((((((((((((((((((**Bone Density**[MeSH Terms]) OR (Bone Densities[Title/Abstract])) OR (Density, Bone[Title/Abstract])) OR (Bone Mineral Density[Title/Abstract])) OR (Density, Bone Mineral[Title/Abstract])) OR (Bone Mineral Content[Title/Abstract])) OR (Bone Mineral Contents[Title/Abstract])) OR (**Fractures, Bone**[MeSH Terms])) OR (Broken Bones[Title/Abstract])) OR (Bone, Broken[Title/Abstract])) OR (Bones, Broken[Title/Abstract])) OR (Bone Fractures[Title/Abstract])) OR (Spiral Fractures[Title/Abstract])) OR (Torsion Fractures[Title/Abstract])) OR (**bone markers**[Title/Abstract])) OR (procollagen Type I N-terminal peptide[Title/Abstract])) OR (PINP peptide[Title/Abstract])) OR (C-terminal telopeptide of type I collagen[Title/Abstract])) ) OR (**adverse effects**[MeSH Terms])) OR (side effects[Title/Abstract])) OR (adverse events[Title/Abstract]) | 672282 |
| #4 | #1 AND #2 AND #3 | 393 |
| #5 | #4 Filters: Randomized Controlled Trial | 105 |
| **Embase** | | |
| #1 | '**alendronate**'/exp OR '4-amino-1-hydroxybutylidene 1,1-biphosphonate':ab,ti OR 'aminohydroxybutane bisphosphonate':ab,ti OR 'mk 217':ab,ti OR 'alendronate monosodium salt, trihydrate':ab,ti OR 'alendronate sodium':ab,ti OR fosamax:ab,ti OR 'i**bandronic acid**'/exp OR ibandronate:ab,ti OR '1-hydroxy-3-(methylpentylamino)propylidenebisphosphonate':ab,ti OR 'ibandronate sodium anhydrous':ab,ti OR boniva:ab,ti OR 'rpr 102289a':ab,ti OR 'bm 21.0955':ab,ti OR '**zoledronic acid**'/exp OR '2-(imidazol-1-yl)-1-hydroxyethylidene-1,1-bisphosphonic acid':ab,ti OR 'cgp 42446a:ab,ti or zometa':ab,ti OR 'zoledronic acid anhydrous':ab,ti OR zoledronate:ab,ti OR '**etidronic acid**'/exp OR 'hydroxyethylidene diphosphonic acid':ab,ti OR etidronate:ab,ti OR hydroxyethanediphosphonate:ab,ti OR 'etidronate disodium':ab,ti OR 'disodium 1-hydroxyethylene diphosphonate':ab,ti OR hedspa:ab,ti OR 'etidronate, tetrapotassium salt':ab,ti OR **'clodronic acid**'/exp OR 'dichloromethane diphosphonate':ab,ti OR clodronate:ab,ti OR 'clodronate disodium':ab,ti OR bonefos:ab,ti OR '**pamidronate**'/exp OR 'amino-1-hydroxypropane-1,1-diphosphonate':ab,ti OR amidronate:ab,ti OR '1-hydroxy-3-aminopropane-1,1-diphosphonic acid':ab,ti OR 'pamidronate disodium':ab,ti OR '**tiludronic acid**'/exp OR '(4-chlorophenyl)thiomethylene bisphosphonic acid':ab,ti OR 'tiludronate disodium':ab,ti OR cl2smbp:ab,ti OR '**6-amino-1-hydroxyhexane-1,1-diphosphonate**'/exp OR neridronate:ab,ti OR 'aminohexane bisphosphonate':ab,ti OR '6 ahhdp':ab,ti OR 'neridronic acid':ab,ti OR '**risedronic acid**'/exp OR '1-hydroxy-2-(3-pyridyl)ethylidene diphosphonate':ab,ti OR 'risedronate sodium':ab,ti OR '2-(3-pyridinyl)-1-hydroxyethylidenebisphosphonate':ab,ti OR risedronate:ab,ti OR 'bisphosphonate risedronate sodium':ab,ti OR 'risedronic acid, monosodium salt':ab,ti | 48387 |
| #2 | Osteopenia:ab,ti OR Osteopenias:ab,ti OR 'Low Bone Density':ab,ti OR 'Bone Density, Low':ab,ti OR 'Low Bone Densities':ab,ti | 18383 |
| #3 | '**fractures, bone**'/exp OR 'broken bones':ab,ti OR 'bone, broken':ab,ti OR 'bones, broken':ab,ti OR 'bone fractures':ab,ti OR 'spiral fractures':ab,ti OR 'torsion fractures':ab,ti OR '**bone density**'/exp OR 'bone densities':ab,ti OR 'density, bone':ab,ti OR 'bone mineral density':ab,ti OR 'density, bone mineral':ab,ti OR 'bone mineral content':ab,ti OR 'bone mineral contents':ab,ti OR 'bone markers':ab,ti OR 'procollagen type i n-terminal peptide':ab,ti OR 'pinp peptide':ab,ti OR 'c-terminal telopeptide of type i collagen':ab,ti OR **'****adverse effects**'/exp OR 'side effects':ab,ti OR 'adverse events':ab,ti | 1660400 |
| #4 | #1 AND #2 AND #3 | 1188 |
| #5 | #4 AND [humans]/lim AND [clinical study]/lim | 138 |
| **Cochrane Library** | | |
| #1 | MeSH descriptor: [Alendronate] explode all trees | 767 |
| #2 | (fosamax):ti,ab,kw OR (aminohydroxybutane bisphosphonate):ti,ab,kw OR (mk 217):ti,ab,kw OR (alendronate monosodium salt, trihydrate):ti,ab,kw OR (alendronate sodium):ti,ab,kw (Word variations have been searched) | 222 |
| #3 | MeSH descriptor: [Ibandronic Acid] explode all trees | 206 |
| #4 | (ibandronate):ti,ab,kw OR (bm 21.0955):ti,ab,kw OR (ibandronate sodium anhydrous):ti,ab,kw OR (boniva):ti,ab,kw OR ('rpr 102289a):ti,ab,kw | 452 |
| #5 | MeSH descriptor: [Zoledronic Acid] explode all trees | 662 |
| #6 | (cgp 42446a):ti,ab,kw OR (zometa):ti,ab,kw OR (zoledronic acid anhydrous):ti,ab,kw OR (zoledronate):ti,ab,kw |  |
| #7 | MeSH descriptor: [Etidronic Acid] explode all trees | 477 |
| #8 | (hydroxyethylidene diphosphonic acid):ti,ab,kw OR (etidronate):ti,ab,kw OR (hydroxyethanediphosphonate):ti,ab,kw OR (etidronate disodium):ti,ab,kw | 297 |
| #9 | MeSH descriptor: [Clodronic Acid] explode all trees | 189 |
| #10 | (dichloromethane diphosphonate):ti,ab,kw OR (clodronate):ti,ab,kw OR (clodronate disodium):ti,ab,kw OR (bonefos):ti,ab,kw | 339 |
| #11 | MeSH descriptor: [Pamidronate] explode all trees | 244 |
| #12 | (amidronate):ti,ab,kw OR (pamidronate disodium):ti,ab,kw | 68 |
| #13 | (tiludronic acid):ti,ab,kw OR (tiludronate disodium):ti,ab,kw OR (cl2smbp):ti,ab,kw | 7 |
| #14 | (neridronate):ti,ab,kw OR (aminohexane bisphosphonate):ti,ab,kw OR (6 ahhdp):ti,ab,kw OR (neridronic acid):ti,ab,kw | 63 |
| #15 | MeSH descriptor: [Risedronic Acid] explode all trees | 259 |
| #16 | (risedronate sodium):ti,ab,kw OR (risedronate):ti,ab,kw OR (bisphosphonate risedronate sodium):ti,ab,kw OR (risedronic acid, monosodium salt):ti,ab,kw | 719 |
| #17 | #1 OR #2 OR #3 OR #4 OR #5 OR #6 OR #7 OR #8 OR #9 OR #10 OR #11 OR #12 OR #13 OR #14 OR #15 OR #16 | 3654 |
| #18 | (Osteopenia):ti,ab,kw OR (Osteopenias):ti,ab,kw OR (Low Bone Density):ti,ab,kw OR (Bone Density, Low):ti,ab,kw OR (Low Bone Densities):ti,ab,kw | 4312 |
| #19 | (fractures, bone):ti,ab,kw OR (broken bones):ti,ab,kw OR (spiral fracture):ti,ab,kw OR (torsion fractures):ti,ab,kw OR (bone, broken):ti,ab,kw | 8635 |
| #20 | MeSH descriptor: [Bone Density] explode all trees | 4829 |
| #21 | (bone densities):ti,ab,kw OR (density, bone):ti,ab,kw OR (bone mineral density):ti,ab,kw OR (bone mineral content):ti,ab,kw OR (density, bone mineral):ti,ab,kw | 13712 |
| #22 | (bone markers):ti,ab,kw OR (procollagen type i n-terminal peptide):ti,ab,kw OR (pinp peptide):ti,ab,kw OR (c-terminal telopeptide of type i collagen):ti,ab,kw | 5779 |
| #23 | (adverse effects):ti,ab,kw OR (side effects):ti,ab,kw OR (adverse events):ti,ab,kw | 328192 |
| #24 | #19 OR #20 OR #21 OR #22 OR #23 | 344599 |
| #25 | #17 AND #18 AND #24 in Trials | 587 |

| **Table S2.** **Risk of bias of randomized controlled trials evaluating bisphosphonates in women with osteopenia** | | | | | | | | | |
| --- | --- | --- | --- | --- | --- | --- | --- | --- | --- |
| **Study, Year** | **Sequence**  **Generation** | **Allocation**  **Concealment** | **Blinding of**  **Participants** | **Blinding of**  **Personnel** | **Blinding of**  **Outcome**  **Assessors** | **Incomplete**  **Outcome**  **Data** | **Selective**  **Outcome**  **Reporting** | **Other**  **Sources**  **of Bias** | **Summary assessments of the risk of bias** |
| **Zoledronate vs. placebo** |  |  |  |  |  |  |  |  |  |
| McClung（2009） | Low risk | Low risk | Low risk | Low risk | Low risk | Low risk | Low risk | Low risk | Low risk |
| Grey(2012) | Low risk | Low risk | Low risk | Low risk | Low risk | Low risk | Low risk | Low risk | Low risk |
| Grey(2017) | Low risk | Low risk | Low risk | Low risk | Low risk | Low risk | Low risk | Low risk | Low risk |
| Reid(2018) | Low risk | Low risk | Low risk | Low risk | Low risk | Low risk | Low risk | Low risk | Low risk |
| **Alendronate vs. placebo** |  |  |  |  |  |  |  |  |  |
| Yen(2000) | Unclear risk | Low risk | Low risk | Low risk | Unclear risk | Low risk | Low risk | Unclear risk | Unclear risk |
| Quandt(2005) | Unclear risk | Low risk | Low risk | Low risk | Low risk | Low risk | Low risk | Low risk | Unclear risk |
| Zhou(2020) | Low risk | High risk | High risk | High risk | Low risk | Low risk | Low risk | Unclear risk | High risk |
| **Risedronate vs. placebo** |  |  |  |  |  |  |  |  |  |
| Valimaki(2007) | Unclear risk | Low risk | Low risk | Low risk | Low risk | Low risk | Low risk | Unclear risk | Unclear risk |
| Sestak(2019) | Low risk | Low risk | Low risk | Low risk | Low risk | Low risk | Low risk | Low risk | Low risk |
| **Ibandronate vs. placebo** |  |  |  |  |  |  |  |  |  |
| McClung(2003) | Unclear risk | Low risk | Low risk | Low risk | Low risk | Low risk | Low risk | Low risk | Unclear risk |
| McClung(2009) | Unclear risk | Low risk | Low risk | Low risk | Low risk | Low risk | Low risk | Low risk | Unclear risk |

| **Table S3*.* Summary of effects of the zoledronate, risedronate, alendronate and ibandronate on BMD, fractures and bone markers in women with osteopenia** | | | | | |
| --- | --- | --- | --- | --- | --- |
| **Comparison** | **Studies, n** | **Participants, n** | **Overall Risk of Bias** | **Consistency** | **Summary of Findings** |
| **BMD** | | | | | |
| Zoledronate vs. Placebo | 4 RCTs | 2517 | Low risk | Results were consistent | Zoledronate significantly increased BMD of the lumbar spine and hip |
| Risedronate vs. Placebo | 2 RCTs | 271 | Low risk | Results were consistent | Risedronate significantly increased the BMD of the lumbar spine |
| Alendronate vs. Placebo | 1 RCTs | 46 | High risk | NA | Alendronate significantly increased the BMD of the lumbar spine |
| Ibandronate vs. Placebo | 2 RCTs | 368 | Low risk | Results were consistent | Ibandronate significantly increased the BMD of the lumbar spine |
| **Fracture risk** |  |  |  |  |  |
| Zoledronate vs. Placebo | 1 RCT | 1505 | Low risk | NA | Zoledronate had a lower risk of fragility fractures (hazard ratio, 0.63), clinical verteral fracture (hazard ratio, 0.41), radiographic vertebral fracture (hazard ratio, 0.60) |
| Alendronate vs. Placebo | 2 RCTs | 3859 | High risk | Results were consistent | Alendronate had a lower risk of fragility fractures (hazard ratio, 0.4), clinical verteral fracture (hazard ratio, 0.46), radiographic vertebral fracture (hazard ratio, 0.64) |
| **Bone markers** | | | | | |
| Zoledronate vs. Placebo | 4 RCTs | 499 | Low risk | Results were consistent | Serum concentrations of β-CTX and PINP were significantly decreased relative to baseline in both zoledronate treatment groups relative to placebo at all timepoints |
| Alendronate vs. Placebo | 1 RCT | 123 | High risk | NA | Serum concentrations of β-CTX and PINP were significantly decreased relative to baseline in both alendronate treatment groups relative to placebo at all timepoints |

**Figure S1**. Meta-analysis of the effects of bisphosphonates on BMD at three skeletal sites weighted mean difference in (A) trochanter BMD, (B) femoral neck BMD, and (C) total body BMD. BMD: bone mineral density.


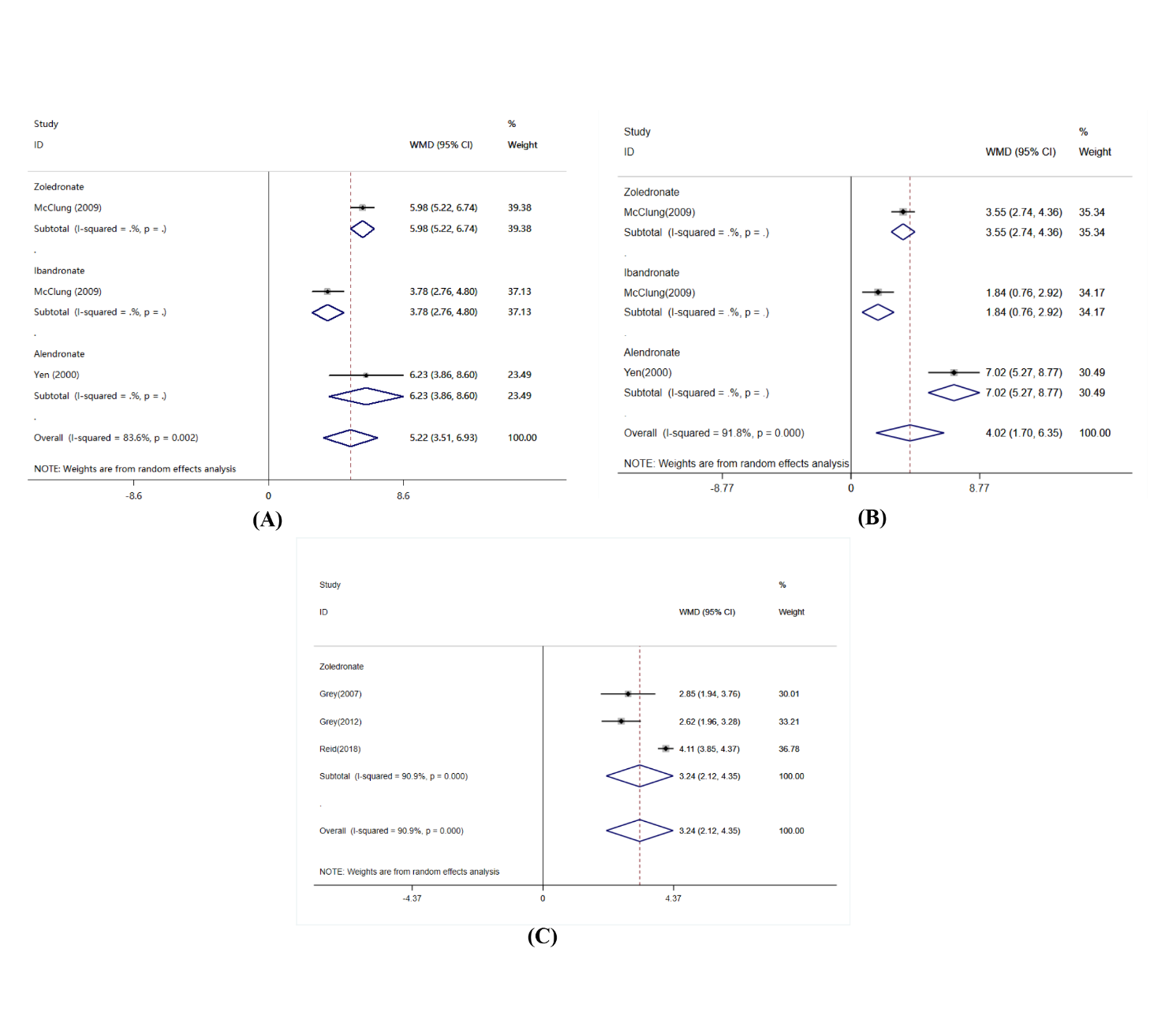


**Figure S2**. Sensitivity analysis for the lumbar spine BMD meta-analysis.


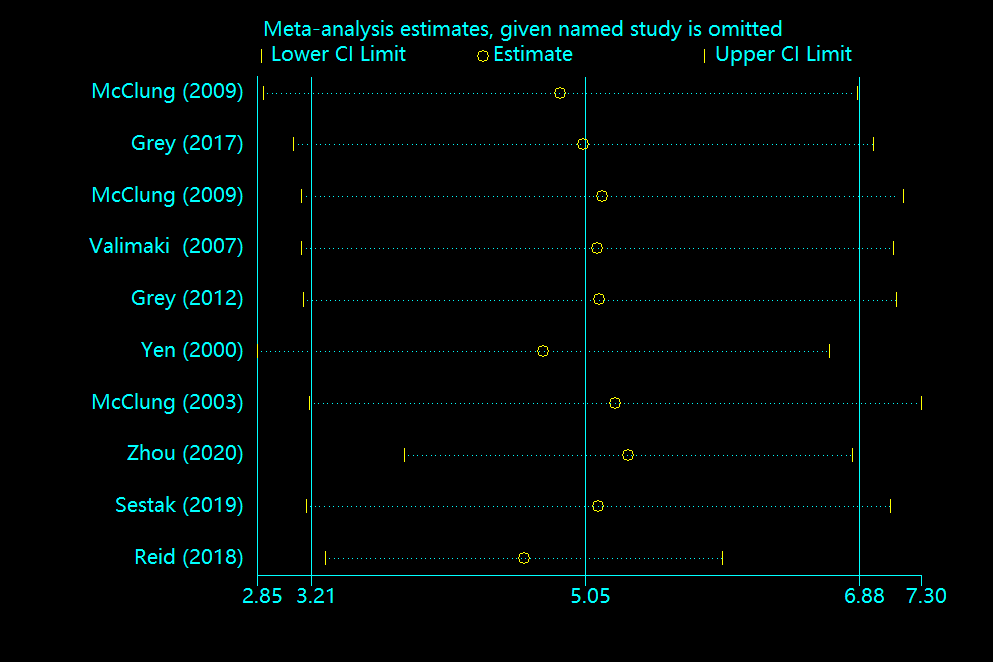


**Figure S3.** Sensitivity analysis for the hip BMD meta-analysis.


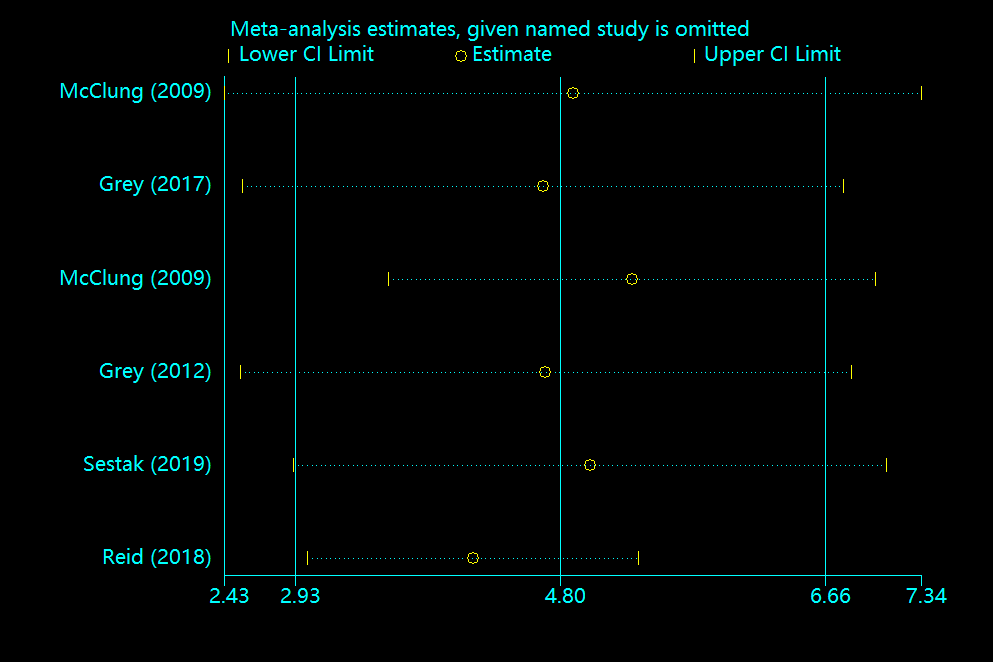


**Figure S4.** Sensitivity analysis for the trochanter BMD meta-analysis.


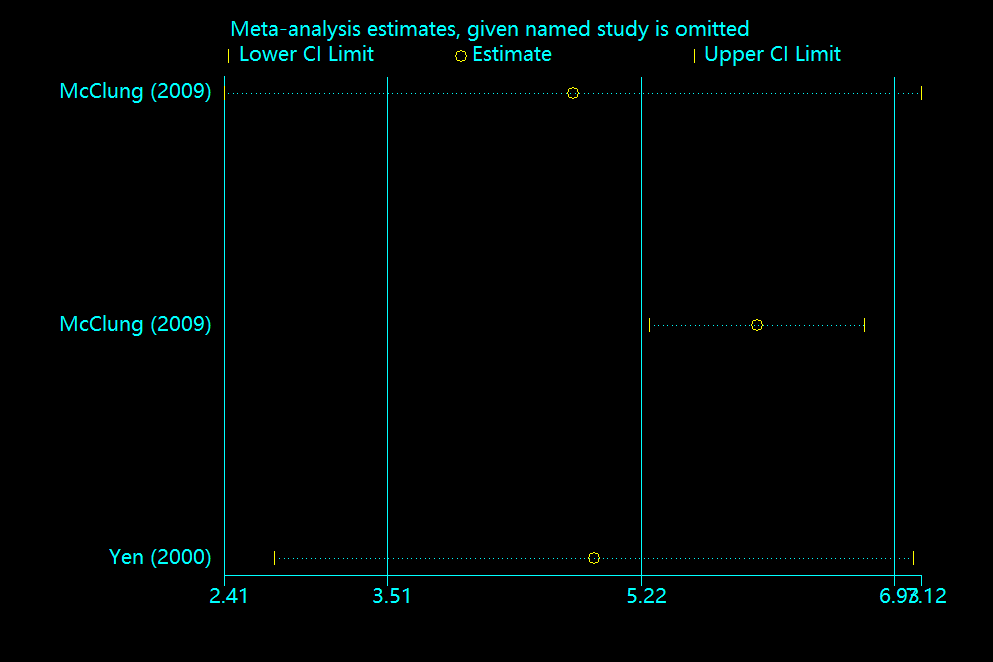


**Figure S5.** Sensitivity analysis for the femoral neck BMD meta-analysis.


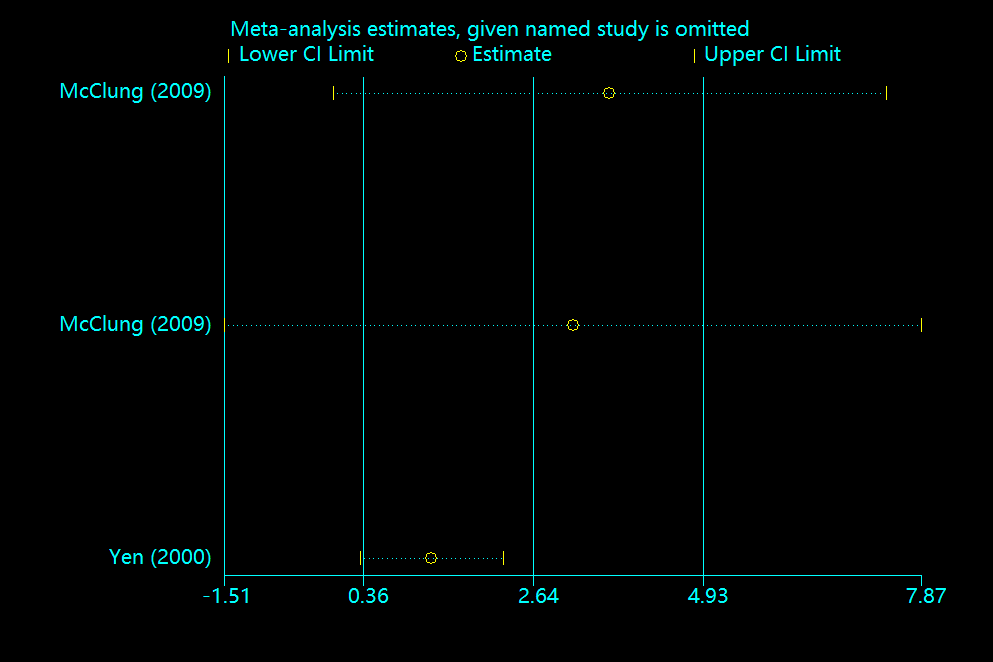


**Figure S6.** Sensitivity analysis for the total body BMD meta-analysis.


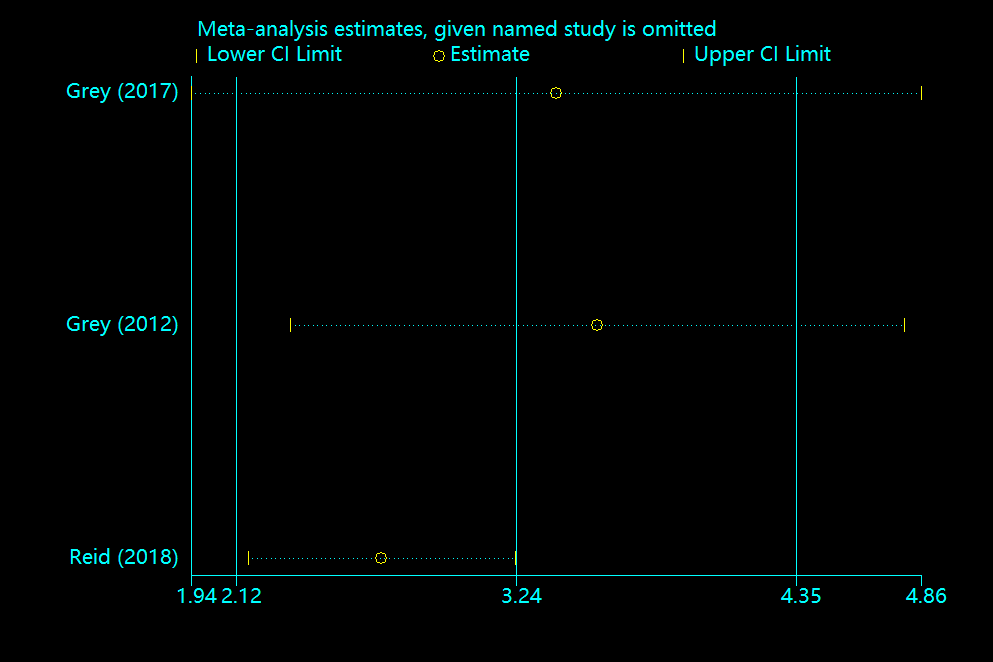


**Figure S7.** Sensitivity analysis for the PINP meta-analysis


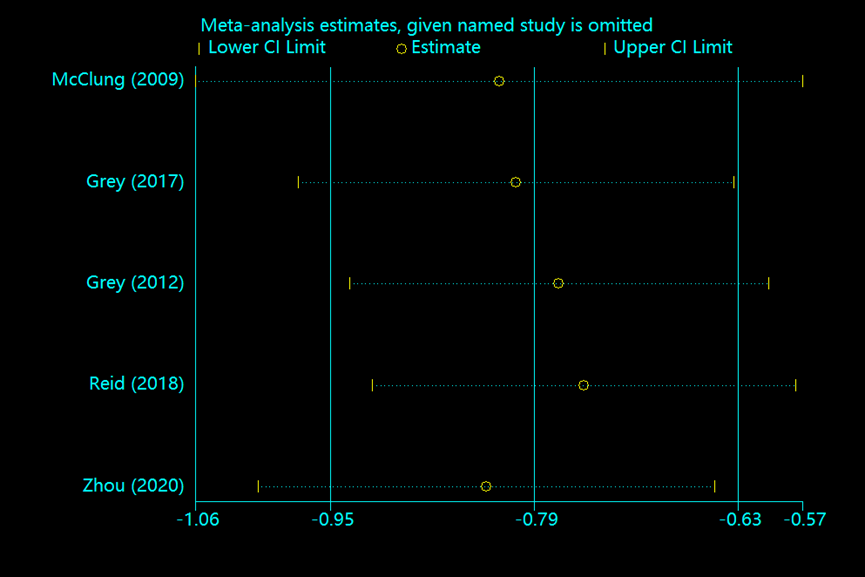


**Figure S8.** Sensitivity analysis for the CTX meta-analysis.


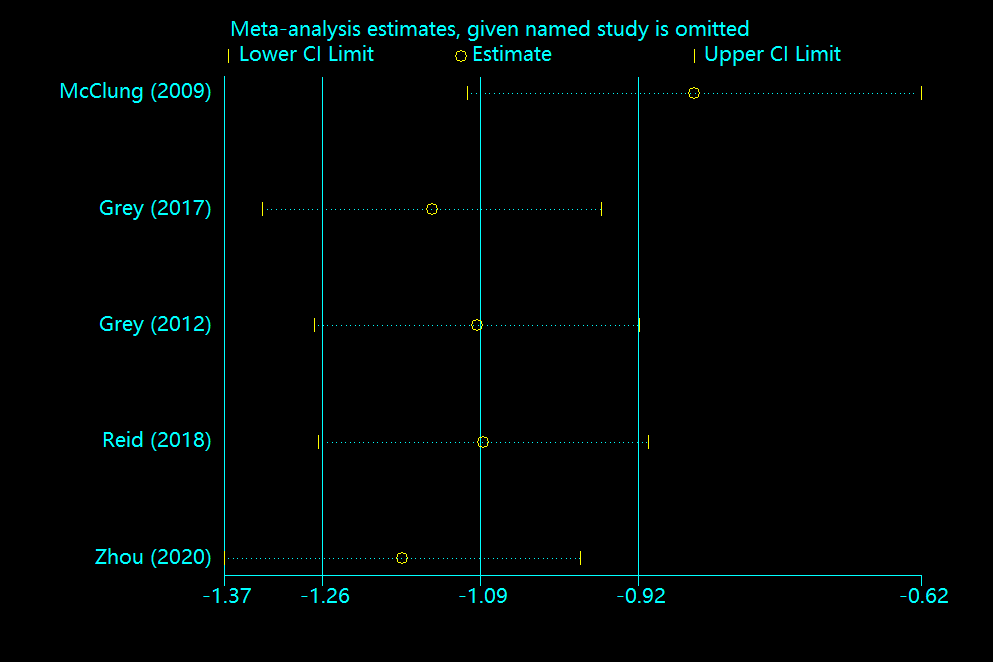

Supplement: Supplementary file 1 [file DataSheet2.docx]
